# In Silico Prediction of Maize microRNA as a Xanthine Oxidase Inhibitor: A New Approach to Treating Hyperuricemia Patients

**DOI:** 10.3390/ncrna11010006

**Published:** 2025-01-15

**Authors:** Manas Joshi, Mohd Mabood Khan

**Affiliations:** 1 Department of Zoology, University of Lucknow, Lucknow 226007, India; 2 Department of Medicine, Vanderbilt University Medical Centre, Nashville, TN 37232, USA

**Keywords:** maize miRNAs, xanthine oxidase, hyperuricemia, base variation

## Abstract

Introduction: Hyperuricemia is characterized by increased uric acid (UA) in the body. The ability to block xanthine oxidase (XO) is a useful way to check how different bioactive molecules affect hyperuricemia. Previous reports showed the significant effect of corn against hyperuricemia disorder with its anti-XO activity. The identification of stable Zea mays miRNA (zma-miR) in humans has opened up a new avenue for speculation about its part in regulating novel human gene targets. Aims: The aim of this study was to investigate the prospects of zma-miRs in XO gene regulation, the possible mechanism, and the interaction analysis of the zma-miR-XO mRNA transcript. Method: Significant features of miRNA-mRNA interaction were revealed using two popular miRNA target prediction software—intaRNA (version 3.3.1) and RNA hybrid (version 2.2.1) Results: Only 12 zma-miR-156 variants, out of the 325 zma-miR’s sequences reported in the miRNA database, efficiently interact with the 3′UTR of the XO gene. Characteristics of miRNA-mRNA interaction were as follows: the positioning of zma-miR-156 variants shows that they all have the same 11-mer binding sites, guanine (G), and uracil (U) loops at the 13th and 14th positions from the 5′ end, and no G: U wobble pairing. These factors are related to the inhibition of functional mRNA expression. Additionally, the zma-miR-156 variants exhibit a single-base variation (SBV), which leads to distinct yet highly effective alterations in their interaction pattern with the XO mRNA transcript and the corresponding free energy values. Conclusion: Therefore, we propose that zma-miR-156 variants may be a promising new bioactive compound against hyperuricemia and related diseases.

## 1. Introduction

High levels of uric acid in the blood characterize hyperuricemia (HUA), a chronic disease commonly known as gout. This process also leads to the deposition of sodium urate crystals in tissues, particularly in the kidneys and joints, resulting in a condition with severe pain and swelling known as gouty arthritis [1,2]. The emerging HUA treatment includes UA synthesis inhibitors and excretion promoters. Other treatment includes urate-lowering therapies, biological uricases (pegloticase and rasburicase), and Interleukin-1 (IL-1) inhibitors [3,4]. Xanthine oxidase (XO) is a conserved enzyme found in many species, including humans. Its sites include a variety of tissues and body fluids, including the vascular endothelium, the liver, the intestine, the kidney, lactating mammary epithelial cells, and the skin. In humans, XO is an important rate-limiting enzyme in purine catabolism, facilitating the oxidation of hypoxanthine to xanthine and then to uric acid. Hydrogen peroxide and reactive oxygen species (ROS) such as superoxide anions are byproducts of this process. Hyperuricemia, and hence gouty arthritis, can result from tissue xanthine oxidase overexpression. In addition, many clinical disorders, like cancer, diabetes mellitus, cardiovascular disease, etc., can be caused by excess ROS production [5,6]. XO inhibitors are widely used in the treatment of hyperuricemia and its associated clinical diseases [5,7,8,9,10]. Clinical practitioners have used common XO inhibitors, such as allopurinol and febuxostat and Topiroxostat, for several years to treat UA levels [4,11]. Even though these drugs lower UA levels, many of them have serious side effects, like stomach problems, rashes, liver and kidney problems, and hepatotoxicity [12]. Because of the mild-to-severe side effects of synthetic drugs, scientists are now looking into highly effective and low-toxicity XO inhibitors. Plant-derived XO inhibitors (PDXOs) are a new replacement for these synthetic drugs due to their anti-hyperuricemic property with fewer side effects [13,14].

Crop plants have nutritional value and can regulate metabolic rates in both humans and domestic animals. There are reports of crops showing anti-XO activity [15,16,17]. One of the most significant crops globally, maize is rich in many bioactive components, including vitamin C, phytate, anthocyanins, carotenoids, and polyphenolic compounds [18,19,20]. There is research suggesting that the consumption of maize can improve gastrointestinal health and lower the risk of certain cancers, obesity, cardiovascular disease, type 2 diabetes, and other chronic conditions [21,22]. Reports suggest that phenolic compounds in maize play a crucial role in managing human XO activity [16,17]. In addition to secondary metabolites, researchers need to look into the unique function of additional bioactive compounds in maize. The presence of other plant bioactive compounds, such as miRNAs, has raised questions regarding their roles as biological regulators due to their occurrence in mammals (e.g., humans, pigs, and mice) [23,24].

Non-coding RNAs of about 22 nucleotides in length, microRNAs (miRNAs), control post-transcriptional gene expression [25,26,27]. Mature miRNAs associate with Argonaute (AGO) proteins, which enhance their capacity to silence genes post-translationally. The miRNA directs the complex to connect with complementary mRNA targets, while the AGO proteins recruit other proteins that inhibit translation and/or degrade mRNA [28]. Therefore, in order to ensure the precise regulation of individual gene expression, miRNA synthesis needs to be controlled at many levels. RBPs, or RNA-binding proteins, play a crucial role in miRNA synthesis and often have conserved RNA-binding domains that can recognize specific RNA sequences or secondary structures [29,30]. RBPs refine miRNA expression through many biogenesis stages, including nuclear processing, nuclear export, cytosolic processing, and loading miRNAs into the RISC complex. Interestingly, there are reports showing the cross-species regulatory mechanism of miRNA in insects, like honey bee caste differentiation [31] and Cuscuta campestris parasitism with host plants [32].

A variety of plant biological processes rely on miRNAs for proper development, stress response, and metabolism [33,34]. Because of their ability to regulate plant–microbe and plant–animal interactions, plant miRNAs are particularly important in cross-kingdom communication [16]. Pathogens can take up plant miRNAs, inhibiting their virulence, whereas intestinal bacteria can absorb them, influencing the composition, localization, and metabolite production of the intestinal microbiota [35,36]. Ingesting pl-miRs has several potential health benefits and exerts multiple biological functions, including antiviral, antitumor, immunomodulatory, anti-inflammatory, and anti-apoptotic effects [16,35,37,38,39,40,41,42]. In addition, plant miRNAs help in fighting cancer by increasing apoptosis and stopping the growth of human tumor cells [38,43,44,45]. pl-miRNAs can also regulate the expression of metabolic genes in the intestinal cells and hepatocytes, leading to reduced lipid accumulation [46,47].

Additionally, some investigations have shown that exogenous miRNAs can be transferred between biological kingdoms via body fluids [47,48,49]. While individual tolerance to intestinal transit varies, pl-miRs are believed to withstand the harsh acidic conditions of the stomach, fluctuating temperatures, and nuclease enzymes. These bioactive molecules can be linked to various RBPs [47,50] or integrated into exosomes [47] or exosome-like nanoparticles, ENLs [51]. Additionally, experimental studies have shown that mRNAs with higher degrees of secondary structure in their untranslated regions (UTRs) are less efficiently targeted by miRNAs and serve as barriers to miRNA-mediated gene silencing [52,53]. Subsequently, these can influence the interior systems of diverse animals.

Previous studies have suggested that maize-derived miRNAs can pass through the gastrointestinal tract of pigs and also modulate mammalian gene expression [54,55]. Li et al. (2019) showed the in vitro regulation of intestinal cell proliferation by zma-miR-156 targeting Wnt10b [54]. This research provided the first evidence that zma-miRNAs could affect the maturation of the intestinal tract and the proliferation of intestinal epithelial cells. These reports add to the growing body of research suggesting that microRNAs found in crops could hold the key to curing human illness through nutritional therapy. Although plant pl-miRs can regulate gene expression, it is important to correctly identify target genes and properly prevent their negative effects [54]. Bioinformatics predictions serve as an initial step in indicating the potential target of plant microRNAs (miRNAs) in humans and other animals. Therefore, there is a need to investigate the influence of zma-miRs on human XO gene targets. Thus, this is the first in silico study to determine the potential function of zma-miRs in XO gene regulation and to analyze the interaction between zma-miRs and XO mRNA transcripts. Consequently, these pl-miRs could represent a starting point in the development of alternate therapeutics to treat HUA and other related diseases.

## 2. Material and Method

### 2.1. In Silico Retrieval of zma-miRs Sequences from miRNA Database

The construction of miRbase web-based miRNA databases has provided large number of published miRNA sequences and annotations [56]. The mature sequences of zma-miRs reported in the miRNA database (http://www.mirbase.org, accessed on 18 October 2024) were retrieved to search for effective and potential XO-mRNA transcript regulators.

### 2.2. Retrieval of 3′UTR of Target Xanthine Oxidase (XO) Gene

Eukaryotic mRNAs contain 5′ and 3′ untranslated regions and play an important role in the post-transcriptional regulation of gene expression by modulating nucleocytoplasmic mRNA transport, translation efficiency, subcellular localization, and message stability. Most miRNAs primarily attach to the 3′ UTR to regulate gene expression. We retrieved the 3′UTR sequence of the XO gene (Gene ID: ENSG00000158125) from the UTR database. It is a database of 5′ and 3′ untranslated sequences of eukaryotic mRNAs, derived from multiple primary data sources and accessible at https://utrdb.cloud.ba.infn.it/utrdb/index_107.html, accessed on 18 October 2024.

### 2.3. Silencing Efficacy of zma-miRs with 3′UTR of XO Genes

IntaRNA is a free miRNA-mRNA target prediction algorithm available on the website (http://rna.informatik.unifreiburg.de/IntaRNA, accessed on 18 October 2024). This online tool was used for the analysis of zma-miR interaction with the 3′UTR of the human XO gene according to two rules. (A) The hybrids must have perfect base pairing between the seed regions (2–8 bases of the mature miR156 variants). (B) The hybrids should meet the following requirements: hybridization energy threshold ≤ −25 kcal/mol and maximum bulge/internal loop length ≤ 5 nt [57]. To further verify the intaRNA prediction result, the analysis software of RNA Hybrid (https://bibiserv.cebitec.uni-bielefeld.de/rnahybrid, accessed on 18 October 2024) was used to predict mRNA targets corresponding to identified miRNAs based on customized parameters [58]. In this prediction, we analyzed zma-miRNA using the 3′UTR of the human XO gene to estimate the energy of association, which includes the minimum free energy and hybridization pattern. This program used several common criteria to determine whether a transcript was a target for zma-miRs, as described previously [47].

### 2.4. Sequence Homology of Selected zma-miR

The relationship between the selected sequence zma-miR targeting the 3′UTR of the human XO gene transcript was analyzed and the number of aligned RNA sequences was calculated using the T-coffee program [59] generated server from the Swiss Institute of Bioinformatics server (http://tcoffee.crg.cat/apps/tcoffee/do:rcoffee, accessed on 18 October 2024).

## 3. Results

### 3.1. zma-miR-156 Variants Target 3′ UTR of Human Xanthine Oxidase Gene

Analysis revealed that of the 325 *ZMA* miRNAs listed in the miRNA database, only 12 zma-miR-156 (zma-miRNA-156) variants showed highly potent interactions with the 3′UTR of the XO gene sequence. IntaRNA analysis revealed that 10 of these 12 zma-miRNA-156 variants (zma-miR-156d-5p, zma-miR-156l-5p, zma-miR-156c, zma-miR-156g-5p, zma-miR-156e-5p, zma-miR-156h-5p, zma-miR-156i-5p, zma-miR-156b-5p, zma-miR-156a-5p, and zma-miR-156f-5p) showed almost identical types of interactions and highly favorable binding, with a hybridization energy of −26.48 kcal/mol. These miRNA and mRNA interactions displayed a perfect pairing of 11-mer binding starting from the second nucleotide at the 5′ end of the zma-miR. The absence of G: U wobble pairing was noted, which is typically associated with the suppression of functional mRNA expression. In silico analysis also revealed miRNA-mRNA binding sequences of 5′GACAGAAGAGAGUGAGC3′, with guanine (G) and uracil (U) loop at the 13th and 14th positions from the 5′ end. Furthermore, zma-miR-156j interaction showed a shift in hybridization energy from −26.48 kcal/mol to −29.98 kcal/mol, with a single G- nucleotide gap at the 11th position. Similarly, zma-miR156k showed a change in hybridization energy from −26.48 kcal/mol to −26.88 kcal/mol (Table 1). For further validation, RNA hybrid software, a tool widely used for miRNA target prediction, showed highly effective interactions with zma-miR156d-5p, zma-miR156l-5p, zma-miR156c, zma-miR156g-5p, zma-miR156e-5p, zma-miR156h-5p, zma-miR156i-5p, zma-miR156b-5p, zma-miR156a-5p, and zma-miR156f-5p, with MFE values of −35.6 kcal/mol. The two zma-miR-156 variants zma-miR156j and zma-miR156k demonstrated a shift, with MFE values of −34.6 kcal/mol and −30.1 kcal/mol, respectively. The variations in free energy scores were −30.1, −34.6, and −35.6 kcal/mol, as shown in Table 2.

### 3.2. Base Variation in zma-miR-156 Variants and Silencing Potency

**Figure 1:**
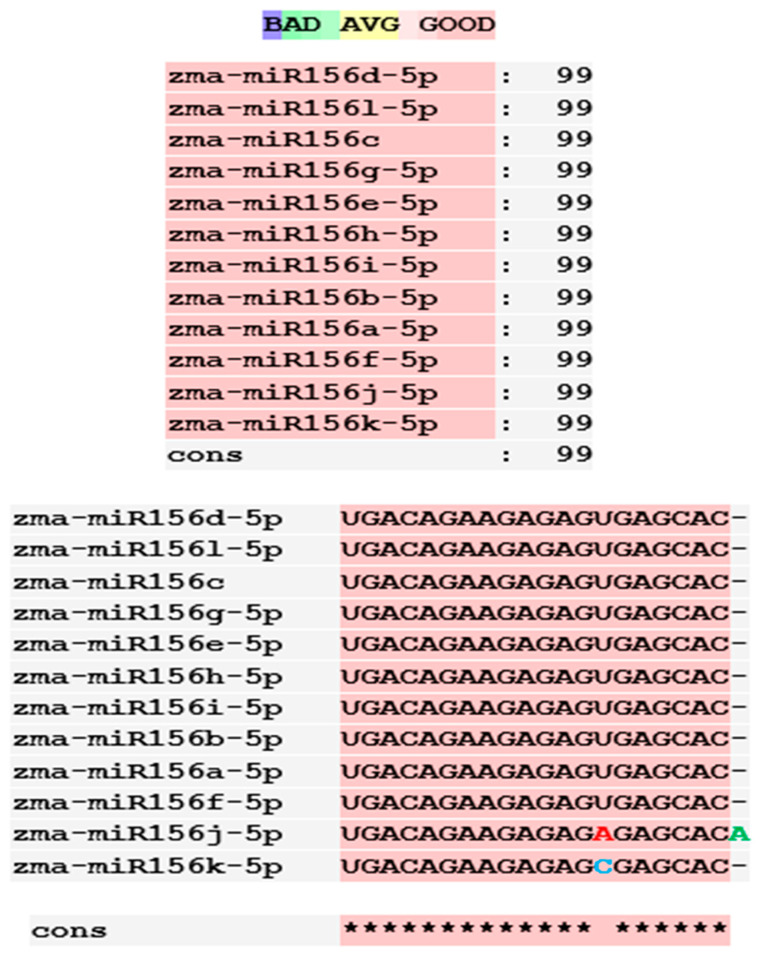
Sequence alignment figure of zma-miR-156 variants generated by the T-coffee alignment program. Nucleotide differences in zma miR 156 variants from conserved sequence are indicated in red, and the extra nucleotide is indicated in green.

The twelve zma-miR156 variants were examined for their variation types and their influence on the silencing efficacy of these variants on the 3′ UTR XO transcript. T-Coffee Alignment, a multiple-sequence alignment software, displayed ten of the twelve miRNA156 variants (zma-miR156d, zma-miR156l, zma-miR156c, zma-miR156g, zma-miR156e, zma-miR156h, zma-miR156i, zma-miR156b, zma-miR156a, and zma-miR156f) with an identical 20-nucleotide sequence of 5′UGACAGAAGAGAGUGAGCAC3′ and showed 100% similarity. These ten sequences indicated a highly favorable interaction with the 3′ UTR of the XO gene, with a hybridization energy of −26.48 kcal/mol and an MFE value of −35.6 kcal/mol. This is followed by a loop of guanine (G) and Uracil (U) nucleotide at the 13th and 14th positions from the 5′ end of zma-miR-156 variants. Two zma-miRNA156 variants displayed single-base variations (SBVs) with ten other zma-miR-156 variants. The zma-mir156j variant showed a change from U to Adenine (A), while zma-mir156k exhibited a U-to-Cytosine (C) substitution at the 14th position from the 5′ end. In addition, zma-miR156j had an extra A base at the 21st nucleotide position of the 5′ end, as illustrated in Figure 1. 

IntaRNA interaction analysis demonstrated that this SBV in zma-miR156j resulted in the alteration of the binding site sequence and hybridization energy from −26.48 kcal/mol to −29.98 kcal/mol, with a single G nucleotide gap at the 11th position. Similarly, the U to C substitution in zma-miR156k modified the hybridization energy from −26.48 kcal/mol to −26.88 kcal/mol. RNA hybrid software further confirmed these findings by showing the MFE value altered from −35.6 to −34.6 kcal/mol in the case of zma-miR156j. In contrast, zma-miR156k resulted in a more significant yet effective shifting of the MFE value from −35.6 to −30.1 kcal/mol.

## 4. Discussion

The search for effective alternatives to the existing medications used in the prevention and treatment of hyperuricemia is of great urgency [60,61]. XO, a potential therapeutic target for HUA, is thought to be responsible for the purine metabolism-related generation of UA and superoxide radical (O^2−^) [62]. UA synthesis is reduced by XO inhibitors (Allopurinol, febuxostat), and the excretion of urea is increased by uricosuric medicines (uricase) as part of urate-lowering therapy [3,4]. New, safer XO inhibitors derived from natural sources are the focus of intense research to address the adverse effects, safety concerns, and tolerability issues that persist with certain existing HUA treatments.

PDXO inhibitors have emerged as a prominent research focus in pharmaceuticals due to the elevated safety of these natural bioactive compounds [34]. Researchers have reported that bioactive compounds such as polyphenols, in crop plants like maize and whole grain wheat, exhibit XO inhibitory activity [15,16,17]. Apart from secondary metabolites, there is a requirement for other bioactive compounds with enhanced efficacy and reduced side effects. The presence of pl-miR in the urine, feces, serum, and tissues of mammals like mice, pigs, and humans has led us to investigate their potential new roles in humans [16,23].

The preliminary phase of the search for probable targets of pl-miRs in humans and other animals employs bioinformatics target prediction tools. Based on this, our study investigates the prospects of zma-miRs as a potential XO gene regulator. The in silico study utilizes two prominent miRNA-mRNA target prediction software packages (RNA hybrid and IntaRNA). The target prediction analysis showed that only 12 of the 325 zma-miRs present in the miRNA database displayed excellent interaction with the 3′UTR of the human XO mRNA transcript. IntaRNA and RNA-hybrid tools show that the pl-miRs demonstrate the following characteristics: The seed region (2–8 nucleotides) exhibits complete complementarity, with an 11-mer match starting from the second nucleotide of the 5′ terminus. Furthermore, the zma-miR-156 variants exhibited SBV, which led to alterations and stable interactions with the 3′ UTR XO mRNA transcript. Table 1 and Table 2 illustrate a structural alteration in the binding of the zma-miR 156 variants with the 3′ UTR XO mRNA duplex. Further inspection of the sequences of zma-miR-156 variants by T-coffee alignment software shows that, out of the twelve miRNA156 variants, ten variants share identical sequences of 5′UGACAGAAGAGAGUGAGCAC3′ (zma-miR156d-5p, zma-miR156l-5p, zma-miR156c, zma-miR156g-5p, zma-miR156e-5p, zma-miR156h-5p, zma-miR156i-5p, zma-miR156b-5p, zma-miR156a-5p, and zma-miR156f-5p). This conserved sequence displays a complete sequence match of 20 nucleotides, a 100% similarity score, and a favorable interaction with a hybridization energy of −26.48 kcal/mol and MFE values of −35.6 kcal/mol. Two zma-miR156 variants show SBV. These include zma-miR156j-5p, which shows the SBV of Uracil (U) to Adenine (A) and Uracil to Cytosine (C) at the 14th position from the 5′ end in zma-miR156k-5p. There is also an extra adenine base in the zma-miR156j-5p variant’s last or 21st nucleotide of the 5′ end, as shown in Figure 1. This leads to a differential yet highly effective interaction with zma-miR-156 variants and the 3′ UTR of the XO mRNA transcript, as shown in Table 1 and Table 2. Since miRNAs can regulate mRNAs completely or partially by pairing complementary bases, the general consensus is that miRNAs bind to the 3′ UTRs of target transcripts at conserved binding sites [63]. The target area has a “seed region” that is 2–7 bp long and has Watson–Crick bases that are perfectly matched to the 5′ end of the miRNAs. The complementarity of seed regions is sufficient to allow miRNAs to repress their targets without requiring significant additional base pairing at the 3′ end of miRNAs [64]. Our results show a binding of 2–11 bp with eleven zma-miR-156 variants and 2–12 bp for zma-miR156j. A small loop or bulge of two nucleotides may develop within miRNA-mRNA interaction. This loop may occur due to mismatches or non-complementary bases, frequently seen in the core region of the miRNA. MicroRNAs predominantly target the 3′ untranslated region (3′ UTR) of messenger RNAs (mRNAs). This region encompasses regulatory elements essential to mRNA stability, translation, and localization [65,66]. Nonetheless, miRNA interactions extend beyond the 3′ UTR. Reports indicate that miRNAs can interact with many sections of mRNAs, including the 5′ UTR, coding sequence, and gene promoters, thereby influencing translation activation or transcription regulation [67,68,69,70]. Thus, the results suggest that these zma-miR-156 variants have the potential to regulate the expression of the XO gene transcript by binding to the 3′ UTR.

**Figure 2:**
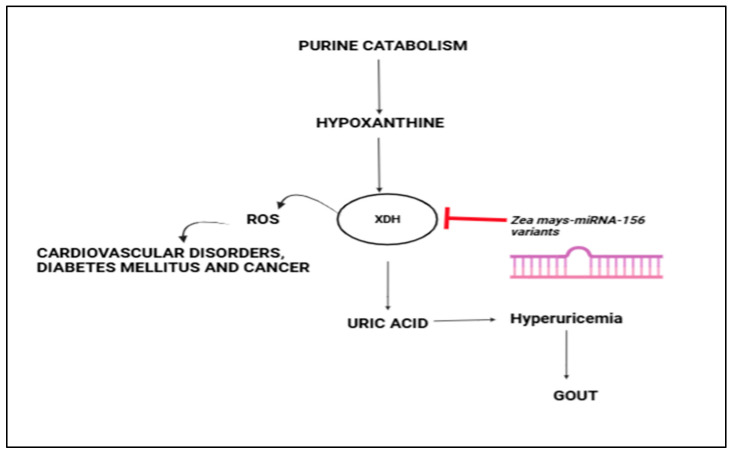
Diagrammatic view of the xanthine oxidase pathways and mode of action of the zma-miRNA 156 family.

There have been prior reports on zma-miRs and their cross-kingdom regulation. One study found that maize-derived miRNAs could cross the gastrointestinal tract and enter the bloodstream of pigs, potentially influencing gene expression [55]. This indicates that pl miRs, including those derived from maize, may possess regulatory functions in mammals. Li et al. (2019) showed that zma-miR-156 regulates intestinal development in mammals by targeting the Wnt/β-catenin pathway [54]. In silico investigations have also identified the role of zma-miRs in targeting genes in various human processes, including cancer development, neurodegenerative diseases, cardiovascular diseases, diabetes, and autism [71]. This study further extends the possibility of zma-miR-156 variants to regulate human XO mRNA transcripts, adding to the list of examples that are already known. Thus, it might be used as a probable regulator of the purine metabolism pathway, leading to a lower UA level in patients with HUA. Figure 2 illustrates the path of the purine catabolism pathways and the regulatory mechanism of zma-miR 156 variants on the 3′ UTR of the XO gene.

In silico analysis is the starting point for gene repression and regulation studies. But there are limitations to the in silico analysis of miRNA target prediction. Firstly, there can be false-positive results with the miRNA-mRNA target prediction tool [72,73]. Secondly, the biological activity of pl-miRs is influenced by processes that include the gastric environment, which plays a significant role in their functionality [74,75]. There are reports confirming exosome-mediated movement [76], in association with argonaute protein [77], and high-density lipoprotein [78], to lessen the degradation of dietary plant miRNAs [79]. Further research has demonstrated that miRNAs are unable to effectively target mRNAs due to higher degrees of secondary structure in their untranslated regions (UTRs), which in turn prevents miRNA-mediated gene silencing [52,53]. As a result, these can impact the internal systems of various animals. Moreover, an obstacle that must be overcome is that findings suggest that pl-miRs are absorbed through the human diet, and they have been found to resist cooking and digestion processes, reaching the distal segments of the gastrointestinal tract [47]. Also, research shows that the presence of terminal 2-O-methyl modification protects plant miRNA from exonuclease and other enzymes that break down proteins [5]. Studies comparing mice fed with a maize-based diet to those given a maize starch diet have revealed that the intestinal cells can accumulate zma-miR-156 [54]. These studies show a promising new avenue for exploring new aspects of the cross-kingdom regulatory role of these pl-miRs in human gene regulation. So, this computational analysis serves as an initial step in examining zma-miR-156 variants in XO gene suppression control. Though the exact role of zma-miR-156 variants is yet to be established experimentally through in vitro and in vivo studies, with the help of these in silico results, we can speculate that zma-miR-156 variants show a complete seed match and highly favorable thermodynamically stable interactions with the 3′UTR of the XO gene transcript. Further studies are in progress including in vitro and in vivo experimentation to validate the above predicted results. If these in vitro and in vivo analysis investigations verify the above hypothesis, then zma-miR-156 variants could explain the observed XO gene expression changes and how to utilize these zma-miR variants in patients with HUA and associated diseases.

## 5. Conclusions

In summary, the in silico study is preliminary evidence of an entirely new class of XO inhibitors in the form of zma-miR-156 variants that display highly favorable interactions with the 3′ untranslated region (UTR) of the XO mRNA transcript. Therefore, it has the potential to serve as a therapeutic drug for patients with HUA and its related complications.

## Figures and Tables

**Table 1:** The intaRNA interaction analysis of zma-miR-156 variants with the 3′UTR of XO gene; the red letter denotes the variable base in zma miR 156 variants sequences.

zma-miR156 3′UTR XO Interaction by IntaRNA Analysis	
1. UGACAGAAGAGAG**U**GAGCAC Target (top): 3′UTR XO Query(bottom): zma-miR-156d-5p, zma-miR-156l-5p, zma-miR-156c, zma-miR-156g-5p, zma-miR-156e-5p, zma-miR-156h-5p, zma-miR-156i-5p, zma-miR-156b-5p, zma-miR-156a-5p, zma-miR-156f-5p	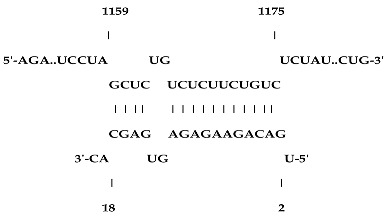 Energy—23.41 kcal/mol Hybridization Energy—26.48 kcal/mol Unfolding Energy—Target: 2.74 kcal/mol Unfolding Energy—Query: 0.33 kcal/mol
2. UGACAGAAGAGAG**A**GAGCACA Target (top): 3UTR XO Query (bottom): zma-mir156j-5p	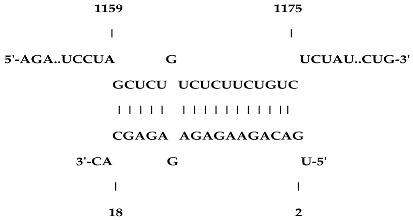 Energy—27.19 kcal/mol Hybridization Energy—29.98 kcal/mol Unfolding Energy—Target: 2.74 kcal/mol Unfolding Energy—Query: 0.05 kcal/mol
3. UGACAGAAGAGAG**C**GAGCAC Target (top): 3UTR XO Query (bottom): zma-miR156k-5p	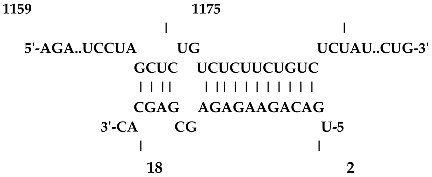 Energy—24.06 kcal/mol Hybridization Energy—26.88 kcal/mol Unfolding Energy—Target: 2.74 kcal/mol Unfolding Energy—Query: 0.08 kcal/mol

**Table 2:** Target interaction analysis of zma-miR-156 variants on the xanthine oxidase gene by RNA hybrid software data showing the minimum free energy values and *p*-values; the red letter denotes the variable base in the zma-miR-156 variants sequences.

Sno	zmamiR 156 Variants’ 3′UTR XO Interaction Detected by RNA Hybrid Analysis		
1	UGACAGAAGAGAG**U**GAGCAC Target (top): 3′UTR XO Query (bottom): zma-miR156d-5p, zma-miR156l-5p, zma-miR156c-5p, zma-miR156g-5p, zma-miR156e-5p, zma-miR156h-5p, zma-miR156i-5p, zma-miR156b-5p, zma-miR156a-5p, zma-miR156f-5p	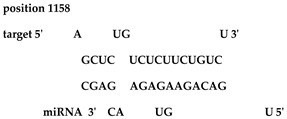 mfe: -35.6 kcal/mol *p*-value: 1.000000e+00	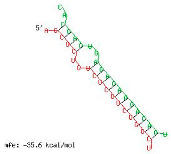
2	UGACAGAAGAG**A**GAGAGCACA Target (top): 3UTR XO Query (bottom): zma-mir156j-5p	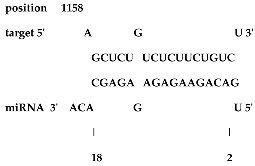 mfe: −34.6 kcal/mol *p*-value: 1.000000e+00	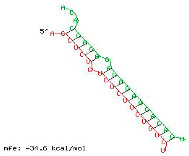
3	UGACAGAAGAGAG**C**GAGCAC Target (top): 3UTR XO Query (bottom): zma-miR156k-5p	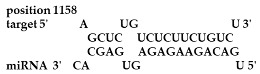 mfe: −30.1 kcal/mol *p*-value: 1.000000e+00	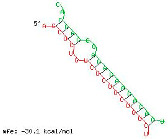

## Data Availability

The data used in the current study are available from the corresponding author upon reasonable request.
